# Supplementary material for: The Role of TSHR, PTEN and RASSF1A Promoters’ Methylation Status for Non-Invasive Detection of Papillary Thyroid Carcinoma
Source: J Clin Med. 2022 Aug 21;11(16):4917. doi: 10.3390/jcm11164917 (PMC9409956; doi:10.3390/jcm11164917)
Supplement: Supplementary file 1 [file jcm-11-04917-s001.zip › jcm-1830961-supplementary.pdf]

Supplementary Table S1. Primers used for quantitative methylation-specific PCR.

Gene Symbol	DNA Strand	Primer ID	Primer Sequence (5′→3′)	Amplicon Size, bp	Reference
RASSF1 A	Antisense	Fwd	GCGTTGAAGTCGGGGTTC	75	[19]
		Rev	CCCGTACTTCGCTAACTTTAAACG		
		Probe	FAM-ACAAACGCGAACCGAACGAAACCA-BHQ1		
PTEN	Sense	Fwd	GTTTCGCGTTGTTGTAAAAGTCG	88	[33]
		Rev	CAATATAACTACCTAAAACTTACTCGAACC		
		Probe	FAM-TTCCCAACCGCCAACCTACAACTACACTTA-BHQ1		
TSHR	Sense	Fwd	GGTGTAGAGTTGAGAATGAGGTGATTTC	122	[34]
		Rev	GCCCAAATCCCTAAACAAATCG		
		Probe	FAM-ACAACACCAACTACAACAAATCCGCCGA-BHQ1		
ACTB	Antisense	Fwd	TGGTGATGGAGGAGGTTTAGTAAGT	133	[19]
		Rev	AACCAATAAAACCTACTCCTCCCTTAA		
		Probe	FAM-ACCACCACCCAACACACAATAACAAACACA-BHQ-1		
